# Adverse effects of total hip arthroplasty on the hip abductor and adductor muscle lengths and moment arms during gait

**DOI:** 10.1186/s13018-020-01832-1

**Published:** 2020-08-12

**Authors:** Xiangjun Hu, Nan Zheng, Wei-Chun Hsu, Jingwei Zhang, Huiwu Li, Yunsu Chen, Kerong Dai, Tsung-Yuan Tsai

**Affiliations:** 1 School of Biomedical Engineering & Med-X Research Institute, Shanghai Jiao Tong University; Engineering Research Center of Digital Medicine and Clinical Translation, Ministry of Education, Shanghai, China; 2 Shanghai Key Laboratory of Orthopaedic Implants & Clinical Translation R&D Center of 3D Printing Technology, Department of Orthopaedic Surgery, Shanghai Ninth People’s Hospital, Shanghai Jiao Tong University School of Medicine, Shanghai, China; 3 Graduate Institute of Biomedical Engineering, National Taiwan University of Science and Technology, Taipei, Taiwan; 4 Department of Orthopaedic, Shanghai Jiao Tong University Affiliated Sixth People’s Hospital, Shanghai, China

**Keywords:** Total hip arthroplasty, Biomechanics, Muscle length, Moment arm, Hip abductor muscle, Hip adductor muscle

## Abstract

**Background:**

Precise evaluation of the hip abductor and adductor muscles function in total hip arthroplasty (THA) patients during gait could help prevent postoperative complications and optimize the rehabilitation training program. The purpose of this study was to elucidate the effects of THA on the hip abductor and adductor muscle lengths and moment arms of in vivo patients during gait.

**Methods:**

Ten unilateral THA patients received CT scans and dual fluoroscopic imaging for the hip kinematics during gait. The hip abductor and adductor muscle insertions were digitized on the 3D hip model for the determination of their dynamic lines of action and moment arms. Changes in the hip abductor and adductor muscle lengths and moment arms of THA patients between the implanted and non-implanted sides were quantified during gait.

**Results:**

The adductor longus, adductor brevis, and pectineus of the implanted hips had significantly (*P* < 0.05) less elongation than that of the non-implanted side during the stance phase. The gluteus medius, gluteus minimus, and piriformis moment arms of the implanted side were significantly shorter. The piriformis muscle moment arm was significantly larger. In the double support phase, the adductor magnus and adductor longus moment arms of the implanted sides were significantly decreased.

**Conclusions:**

Results suggested that the adverse effects of THA on hip stability. Development of a rehabilitation program considering the effects of THA is essential. Accurate surgical techniques may reduce the impact of THA on the peripheral muscles.

## Background

Total hip arthroplasty (THA) is a standard surgical procedure for the treatment of end-stage hip osteoarthritis and avascular necrosis of the femoral head. THA can effectively relieve pain, restore hip function, and improve the quality of life [1]. However, asymmetric gait, pelvic drop, and limping were reported in patients after THA [2]. These dysfunctions can lead to continuous soft tissue contraction around the hip joint, resulting in prosthesis dislocation and loosening, periprosthetic fracture, and unequal lower limb length [3].

Previous studies reported that the hip muscle function could be affected after THA. Demos et al. [4] found that although THA can improve walking function in most patients, 11.6% of patients still suffered from moderate to severe claudication or limping. During the single-leg support period, the trunk swings to the operated leg, and the limp is swaying left and right on the coronal plane due to the lack of hip abductor and adductor muscle strength [5]. Moreover, studies have demonstrated that insufficient muscle strength and tension in the hip abductor and adductor muscles (nerve injury or limb shortening) are the causes of THA dislocation [6, 7]. Rudiger et al. [8] reported that the decrease in the femoral offset after THA would reduce the abductor moment arms during gait using the musculoskeletal model in OpenSim. Precise evaluation of the function of the hip abductor and adductor muscles in THA patients during gait could help prevent postoperative complications and optimize the rehabilitation training program. However, precise biomechanical effects of THA on the hip abductor and adductor muscles of in vivo patients during gait remains unknown.

The dual fluoroscopic imaging system (DFIS)-based tracking technique has been extensively applied to capture the motion of in vivo lower limb joints during gait and allowed accurate quantification of 3D joint kinematics [9–11]. Recently, some researchers have used the DFIS for the measurement of soft tissue length changes during motion [12, 13]. However, it has not been used to quantify the effects of THA on the hip muscle lengths and moment arms. This study aimed (1) to quantify the hip abductor and adductor muscle lengths and moment arms in unilateral THA patients during gait and (2) to analyze the effects of THA on the biomechanical parameters of the hip abductor and adductor muscles.

## Materials and methods

### Subject recruitment and tasks

Our Institutional Review Board approved this study. Ten THA patients (8 women, 2 men; age 61.9 ± 9.4 years) with no history of any surgical and orthodontic treatment were recruited. All the patients were with end-stage hip osteoarthritis before THA. The average follow-up time was 1 year (10.2 ± 1.6 months). Harris hip scores (HHS) of all the patients were higher than 90. These patients received unilateral THA from the same surgeon using the posterior approach. All subjects received a computed tomography (CT) scan (SOMATOM Definition AS1, Siemens), providing an in-plane resolution of 0.98 mm at a 1-mm slice thickness, from the L5 vertebra to the tibial plateau for the construction of surface models of the femur, acetabular cup, pelvis, and prosthesis. The patients performed treadmill walking at self-selected speed under the DFIS surveillance (BV Pulsera, Phillips Medical, USA) for both the implanted and non-implanted hips.

### The 3D skeletal model reconstruction and hip kinematics measurement during gait

We segmented the CT images and reconstructed the 3D surface models of the femoral stem, acetabular cup, pelvis, and prosthesis of both sides using a region growing method (Amira, Thermo Fisher Scientific, Waltham, Massachusetts, USA). The reconstructed 3D surface models were imported into MATLAB (R2018a, MathWorks, Natick, MA, USA) to establish anatomical coordinate systems (ACS) for the pelvis and femur following the recommendation of the International Society of Biomechanics [14].

The DFIS projection geometry was obtained through the system calibration procedure for the creation of a virtual environment in software [15]. The six degrees of freedom (6-DoF) of the hip can be determined by matching the contours of the 3D models, and their fluoroscopic projections [9]. The measurement errors were 0.35 mm and 0.55° in measuring the hip joint translations and rotations, respectively [9].

### Muscle lengths and moment arms quantification

**Fig. 1:**
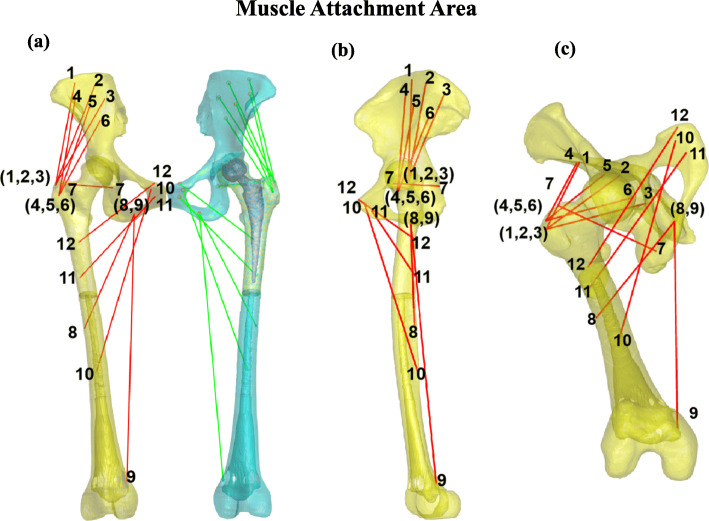
The pelvic and femoral bone models with the hip abductor and adductor muscle lines of action, viewing from the front (**a**), lateral (**b**), and oblique (**c**). The gluteus medius and minimus are divided into anterior, middle, and posterior bundles. The adductor magnus is divided into anterior and posterior bundles. All the muscles are listed as follows: gluteus medius anterior bundle (1, GMDA), gluteus medius median bundle (2, GMDM), gluteus medius posterior bundle (3, GMDP), gluteus minimus anterior bundle (4, GMIA), gluteus minimus median bundle (5, GMIM), gluteus minimus posterior bundle (6, GMIP), piriformis (7, PF), adductor magnus anterior bundle (8, AMA), adductor magnus posterior bundle (9, AMP), adductor longus (10, AL), adductor brevis (11, AB), and pectineus (12, PT)

The 3D skeleton models were imported into MATLAB for the attachment area determination of the hip abductor and adductor muscles in the pelvis and femur of the native side. The muscle attachment sites were digitized following human anatomy studies [16]. We stratified the hip muscles into two groups according to their functions. First, the abductor muscle group includes the gluteus medius (GMD), gluteus minimus (GMI), and piriformis (PF). Second, the adductor muscle group includes the adductor magnus (AM), adductor longus (AL), adductor brevis (AB), and pectineus (PT). If the longitudinal direction of the muscle bundles was straight, the center of the attachment was determined as the starting and ending points to simulate each muscle’s lines of action [17]. If the longitudinal direction of the muscle bundles was curve, the bone structure turning point was taken as the ending point [16]. If the muscle attachment area was wide, we divided it into bundles to reflect the function of different muscle bundles (Fig. 1). To determine the muscle attachments of the implanted side, we mirrored and aligned the non-implanted femur and muscle attachments to the remaining femur of the implanted side [10] (Fig. 1).

The dynamic changes of each muscle length and moment arm during gait were calculated using in-vivo 6-DoF hip joint kinematics. The center of the femoral head was considered as the point of action for the lower limb muscles. Moment arm was defined as the distance from the center of the femoral head to each muscle’s lines of action. The in vivo hip muscle lengths and moment arms data of both the implanted and non-implanted hips against the gait cycle were calculated and compared.

### Statistical analysis

The Wilcoxon signed-rank test was used to determine whether there was a significant difference in hip muscle lengths and moment arms between the implanted and non-implanted sides during gait. A level of significance was set at 0.05. All the statistics were performed in MATLAB. Statistical power analyses were computed in G*power (Franz Faul, Christian-Albrechts-Universität Kiel, Germany).

## Results

### The difference in muscle lengths

**Fig. 2:**
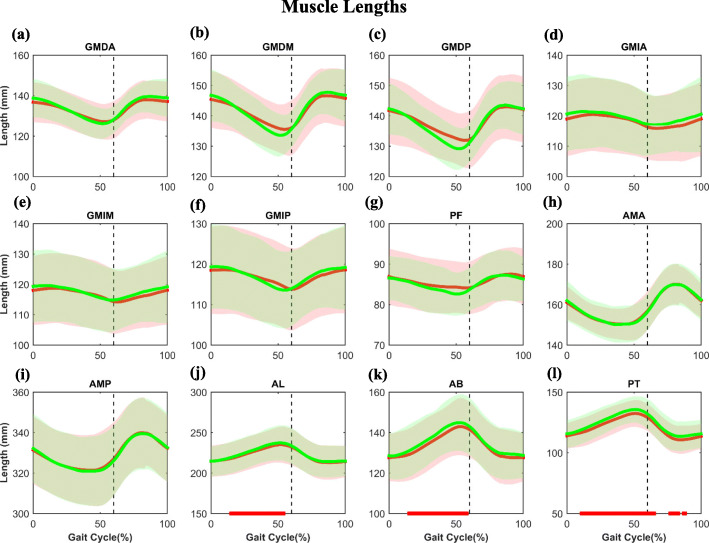
The average and standard deviation of the hip muscle lengths in THA and contralateral non-operated hip are illustrated during gait. The black vertical dashed lines indicate toe-off. The red and green lines represent the implanted and non-implanted sides. Statistical significance between the legs is marked by the bold red line along the *X*-axis of each graph. Muscle abbreviations are in Fig. 1 caption

The adductor muscles of the implanted side were significantly (*P* < 0.05) shorter than that of the contralateral side (Fig. 2j-l) with maximum differences of 2.9 mm, 2.9 mm, and 3.6 mm for the AL, AB, and PT, respectively (Table 1). No significant differences were found in the abductor muscle lengths between the implanted and non-implanted sides during gait (Fig. 2).

**Table 1:** Maximum shortening and elongation in each adductor and abductor muscle of the implanted side relative to the non-implanted side are listed throughout gait

Muscle length	Abductor muscles							Adductor muscles				
	GMD			GMI			PF	AM		AL	AB	PT
	GMDA	GMDM	GMDP	GMIA	GMIM	GMIP		AMA	AMP			
Maximum shortening												
Mean (mm)	−2.0	−1.3	−0.9	−1.9	−1.6	−1.3	−0.6	−0.9	−0.8	−2.9	−2.9	−3.6
SD (mm)	5.2	6.1	6.5	4.3	4.5	4.8	3.6	3.6	3.5	3.7	3.5	4.0
*P* value	0.215	0.648	0.648	0.085	0.372	0.617	0.913	0.286	0.133	0.014*	0.008*	0.007*
Maximum elongation												
Mean (mm)	1.1	2.3	3.4	N.A.	0.5	1.4	1.7	0.6	1.0	0.2	N.A.	N.A.
SD (mm)	4.8	5.3	6.0	N.A.	4.5	4.9	4.3	5.1	5.0	5.0	N.A.	N.A.
*P* value	0.420	0.170	0.078	N.A.	0.586	0.349	0.133	0.811	0.586	0.617	N.A.	N.A.

Muscle abbreviations are in Fig. 1 caption

A Wilcoxon signed-rank test was used to determine whether there is a significant difference

*N.A.* not available

*P value < 0.05

### The difference in muscle moment arms

**Fig. 3:**
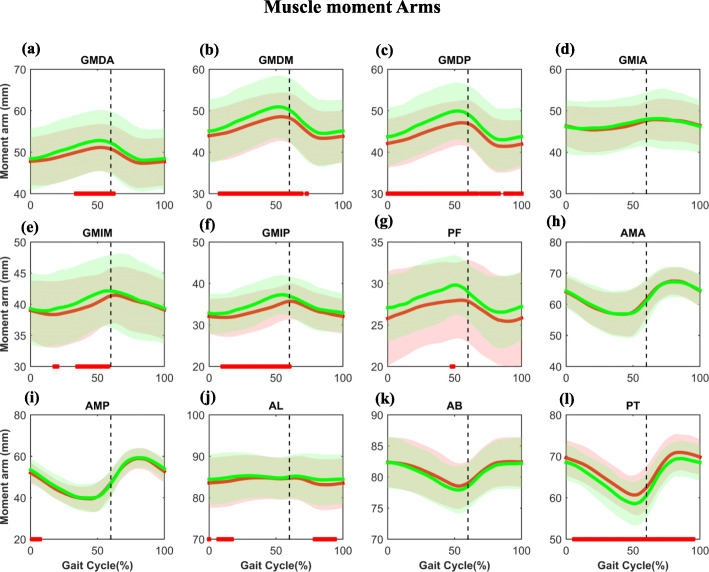
The average and standard deviation of the hip muscle moment arms in THA and contralateral non-operated hip are illustrated during gait. The black vertical dashed lines indicate the end of the stance phase. The red and green lines represent the implanted and non-implanted sides. Statistical significance between the legs is marked by the bold red line along the *X*-axis of each graph. Muscle abbreviations are in Fig. 1 caption

For the abductor muscles in the stance phase during gait, the GMDA, GMIM, and GMIP moment arms of the implanted side were significantly shorter than those of the contralateral side (*P* < 0.05) with the maximum differences of 1.7 mm, 1.5 mm, and 2.5 mm, respectively (Fig. 3a, e-f, Table 2). The GMDM and GMDP of the implanted hips showed significantly shorter moment arms—not only in the stance phase but also in the partial swing phase (Fig. 3b-c) with maximum differences of 2.5 mm and 3.0 mm (Table 2). For the adductor muscle in the double support phase during gait, the AMP (Fig. 3i) and AL (Fig. 3j) moment arms of the implanted sides were significantly shorter than those of the non-implanted sides. Moreover, in the terminal swing phase, a significantly shorter moment arm (*P* < 0.05) (Fig. 3j) was observed in the AL of the implanted side. The maximum differences were 1.5 mm and 1.2 mm (Table 2). The PT moment arm (Fig. 3l) was significantly larger (*P* < 0.05) than that of the non-operated side almost during the entire gait, with maximum difference of 2.2 mm (Table 2).

**Table 2:** Maximum shortening and elongation of muscle moment arm in each adductor and abductor muscle of the implanted side relative to the non-implanted side are listed throughout gait

Muscle moment arm	Abductor muscles							Adductor muscles				
	GMD			GMI			PF	AM		AL	AB	PT
	GMDA	GMDM	GMDP	GMIA	GMIM	GMIP		AMA	AMP			
Maximum shortening												
Mean (mm)	−1.7	−2.5	−3.0	−0.7	−1.5	−2.5	−1.9	−0.5	−1.5	−1.2	N.A.	N.A.
SD (mm)	3.4	3.2	3.2	2.8	2.4	2.4	3.4	2.1	2.3	1.9	N.A.	N.A.
*P* value	0.025*	0.010*	0.008*	0.122	0.022*	0.008*	0.053	0.472	0.048*	0.039*	N.A.	N.A.
Maximum elongation												
Mean (mm)	N.A.	N.A.	N.A.	0.1	N.A.	N.A.	N.A.	0.6	0.5	N.A.	0.8	2.2
SD (mm)	N.A.	N.A.	N.A.	3.1	N.A.	N.A.	N.A.	2.9	4.9	N.A.	1.6	2.1
*P* value	N.A.	N.A.	N.A.	0.811	N.A.	N.A.	N.A.	0.306	0.811	N.A.	0.071	0.005*

Muscle abbreviations are in Fig. 1 caption

A Wilcoxon signed-rank test was used to determine whether there is a significant difference

*N.A.* not available

*P value < 0.05

## Discussion

Statistically significant differences were found in the hip abductor and adductor muscle lengths and moment arms between the legs in unilateral THA patients during gait. Significantly shorter abductor moment arms, less elongated adductor muscle, and longer adductor muscle moment arms during the stance phase of the gait cycle were observed in the implanted side than the contralateral non-implanted side (*P* < 0.05, Tables 1 and 2, Figs. 2 and 3). Among all the hip abductor and adductor muscles, the GMDP and PT moment arms were affected the most with the highest difference during most of the gait cycle (Figs. 2 and 3). The GMDP, AMP, and AL moment arms significantly decreased during the load-bearing response period (Fig. 3). Our findings indicated that THA would change the biomechanical parameters of the hip abductor and adductor muscles, which could result in muscle weakness and limit the effectiveness of regular rehabilitation.

The muscle lengths and moment arms are essential biomechanical parameters for muscle function evaluation. Dostal et al. [17] quantified hip muscle lengths and moment arms in a male cadaver by dissecting the proximal and distal muscle attachments. They reported that the GMD, GMI, and AM anterior bundles provided a relatively larger moment arm in each muscle. A clinical commentary study reviewed the hip muscle function based on a hypothetical model and reported that GMD has the largest abduction moment arm in the abductor muscles [18]. Our results are in line with the abovementioned findings. Further, Bjørdal et al. [19] reported a larger abductor moment arm in the implanted side (65.4 ± 5.9 mm) than in the non-implanted side (58.0 ± 6.6 mm) on radiographs of 148 THA patients. In contrast, we found shorter abductor moment arms after THA. This difference may attribute to different measurement methods, surgical approaches, and implant systems used between studies.

Abductor deficiency has been reported after THA [2, 20]. Previous studies demonstrated that when the hip abductor is weak, the pelvis drops to the sound side, leading to gait instability [3, 7]. In this study, we found that the abductor muscle lengths of the THA side were longer than the contralateral non-implanted side during the support phase (Fig. 2), implying less abductor muscle contraction in the THA side. Furthermore, the moment arms of these abductor muscles were smaller than that of the contralateral sound side (Fig. 3), resulting in reduced abductor moment and muscle efficiency. On the other hand, previous studies reported that many THA patients experienced persistent adductor muscle contracture [21], which collaborates with the significant adductor muscle shortening than that of the non-implanted side in our study (Fig. 2). The abductor muscle weakness and contracture observed in THA patients may relate to the adverse biomechanical effects of THA.

The adductor and abductor muscles also assist hip flexion, extension, and rotation. The AM began to pull at the end of the loading response period. The AM and GMDP help the gluteus maximus muscle in hip extension, according to the previous studies [18]. In addition to the AM and GMDP, AL can also act as a hip flexor and extensor [18]. In this study, we found that AMP, GMDP, and AL of the THA side were shortened during the loading response period, implying concentrically contraction. Additionally, their moment arms are significantly smaller than the non-operated side (Figs. 2 and 3). The GMIP was considered as a secondary external rotator of the hip [18]. In this study, we observed that the GMIP of the THA side had a significantly shorter moment arm, which might associate with the excessive internal rotation reported in THA patients [10]. The adverse biomechanical effects may associate with the hip extensor weakness, lower hip range of motion, and abnormal hip internal rotation during gait (Figs. 2 and 3).

In this study, Harris hip scores (HHS) of all the patients were higher than 90, indicating these THA patients were well-functioning. However, significant changes in hip abductor and adductor muscle lengths and moment arms in the implanted side were observed. In clinical practice, it is challenging to identify the malfunction of each muscle non-invasively. Our study provided a new quantitative method to evaluate the in vivo performance of each muscle during functional activity, which was essential to develop a personalized rehabilitation strategy after surgery for better clinical outcomes.

The Sherrington’s reciprocal inhibition principle states that tensioned or shortened antagonist muscles may reflexively inhibit active muscles [22]. The hip adductor muscle is the antagonist of the hip abductor muscle. If the hip adductor muscle becomes tight and shortened, it may, in turn, be a cause of hip abductor muscle weakness. Our study found that in THA patients, the hip adductors of the operated side were shortened compared with the non-operated side during gait, which may reflexively inhibit the hip abductor muscle. The current rehabilitation training for patients after THA focuses more on strengthening the hip abductor muscles, which may be limited. The previous studies reported asymmetric muscle activities even 2 years after THA [23, 24]. Therefore, the training of antagonist muscles should be enhanced together. We suggest that THA patients should first start to recover the hip adductor muscle tension and length, especially the PT, the most affected adductor, through muscle energy technique [25] and specific fascial release technique. Subsequently, strengthen the weak or inhibited abductor hip muscles, especially the GMD, the most affected abductor.

Janda [26] reported that tensed or shortened antagonist muscles often become active in unrelated movements, further worsening dynamic posture control. This compensation will naturally decrease hip extension and increase hip internal rotation and adduction [10]. In this study, the decreased GMDP, AMP, and AL moment arms after THA may decrease hip extension, and GMIP may increase hip internal rotation during the gait cycle. This compensatory model will lead to the biomechanical changes of the entire lower limb, which in turn will lead to incorrect movements and aggravates injuries [27, 28]. It is suggested that postural control, combined with individualized muscle training, should be implemented in patients after THA to maintain the body’s overall balance by strengthening the coordination and control ability between the muscle groups.

Biomechanical changes in the musculoskeletal system after THA are closely related to surgical techniques and prosthesis design [8]. Accurate surgical techniques as well as optimal prosthesis design and positioning, can reduce the impact of joint replacement on peripheral muscle function. A previous study suggested that accurate intraoperative femoral offset reconstruction is essential for maintaining the abductor muscle moment arm [8]. The displacement of the center of the femoral head may occur due to surgeon preferences (e.g., excessive correction of native femoral anteversion), inaccurate preoperative templating, or limited selection of available implants. Currently, the hip prosthesis design features a smaller femoral head, higher rotation center, wider neck, and lower range of motion, which negatively affects the biomechanical parameters and peripheral muscle performance [29]. We recommend that the next generation hip prosthesis geometry should be optimized for better functional recovery and clinical outcomes.

The present study should be interpreted in light of potential limitations. Only 10 patients who received THA for a year participated. However, using the muscle lengths difference between the implanted and non-implanted sides, the statistical verification power of the data is 97% through G-Power calculation. Future studies should recruit more patients who underwent THA with preoperative data and longer follow-up to compare the effects of component positioning, follow-up times, and rehabilitation program on muscle function recovery.

## Conclusion

This study quantified the in vivo dynamic hip abductor and adductor muscle lengths and moment arms in unilateral THA patients during gait using the DFIS tracking technique. Significantly shorter abductor moment arm, less elongated adductor muscle, and longer adductor muscle moment arm during the stance phase of the gait cycle were observed in the implanted side than the contralateral non-implanted side. We suggest that patients after THA should first recover the normative length of hip adductor muscles, which can be achieved through physiological treatment of muscle energy technique. Moreover, postural control combined with individualized muscle training is recommended in patients after THA to maintain the overall balance of the body by strengthening the coordination and control ability between the abductor and adductor muscle groups. Accurate surgical techniques, as well as optimal prosthesis design and positioning, could reduce the impact of joint replacement on the biomechanical parameters of the peripheral muscles. Further studies are guaranteed to minimize the adverse effects of THA on the surrounding muscles.

## Data Availability

The datasets used and analyzed during the current study are available from the corresponding author on reasonable request.

## Abbreviations

THA: Total hip arthroplasty
DFIS: Dual fluoroscopic imaging system
CT: Computed tomography
6-DoF: Six degrees of freedom
GMD: Gluteus medius
GMDA: Gluteus medius anterior bundle
GMDM: Gluteus medius median bundle
GMDP: Gluteus medius posterior bundle
GMI: Gluteus minimus
GMIA: Gluteus minimus anterior bundle
GMIM: Gluteus minimus median bundle
GMIP: Gluteus minimus posterior bundle
PF: Piriformis
AM: Adductor magnus
AMA: Adductor magnus anterior bundle
AMP: Adductor magnus posterior bundle
AL: Adductor longus
AB: Adductor brevis
PT: Pectineus
